# Deciphering anoikis resistance and identifying prognostic biomarkers in clear cell renal cell carcinoma epithelial cells

**DOI:** 10.1038/s41598-024-62978-0

**Published:** 2024-05-27

**Authors:** Junyi Li, Qingfei Cao, Ming Tong

**Affiliations:** Department of Urology, The First Affiliated Hospital of Jinzhou Medical University, Jinzhou, 121001 Liaoning China

**Keywords:** Cancer, Computational biology and bioinformatics, Biomarkers, Medical research, Oncology, Urology

## Abstract

This study tackles the persistent prognostic and management challenges of clear cell renal cell carcinoma (ccRCC), despite advancements in multimodal therapies. Focusing on anoikis, a critical form of programmed cell death in tumor progression and metastasis, we investigated its resistance in cancer evolution. Using single-cell RNA sequencing from seven ccRCC patients, we assessed the impact of anoikis-related genes (ARGs) and identified differentially expressed genes (DEGs) in Anoikis-related epithelial subclusters (ARESs). Additionally, six ccRCC RNA microarray datasets from the GEO database were analyzed for robust DEGs. A novel risk prognostic model was developed through LASSO and multivariate Cox regression, validated using BEST, ULCAN, and RT-PCR. The study included functional enrichment, immune infiltration analysis in the tumor microenvironment (TME), and drug sensitivity assessments, leading to a predictive nomogram integrating clinical parameters. Results highlighted dynamic ARG expression patterns and enhanced intercellular interactions in ARESs, with significant KEGG pathway enrichment in MYC + Epithelial subclusters indicating enhanced anoikis resistance. Additionally, all ARESs were identified in the spatial context, and their locational relationships were explored. Three key prognostic genes—TIMP1, PECAM1, and CDKN1A—were identified, with the high-risk group showing greater immune infiltration and anoikis resistance, linked to poorer prognosis. This study offers a novel ccRCC risk signature, providing innovative approaches for patient management, prognosis, and personalized treatment.

## Introduction

Renal cell carcinoma (RCC), recognized as the third most common urological malignancy, poses significant challenges in clinical and scientific domains for urologists. In 2020, over 400,000 new RCC cases were documented globally. ccRCC, the predominant subtype of RCC, constitutes about 70–80% of all RCC cases^1,2^. Characterized by high metastasis and mortality rates, ccRCC is increasingly diagnosed in younger populations^3^. In 2022, China reported 77,410 new RCC cases and 46,345 deaths^4^. Although partial and radical nephrectomies are the primary ccRCC treatments, surgical intervention often doesn’t prevent recurrence in early-stage cases^5,6^. The prognosis for ccRCC patients remains relatively poor. Early-stage ccRCC often presents without distinct clinical symptoms, resulting in approximately 30% of patients receiving a diagnosis at advanced stages, characterized by distant metastasis and consequently missing opportunities for surgical intervention^7^. This underscores the critical need for innovative diagnostic and prognostic models, alongside the development of new biomarkers and molecular targets for ccRCC.

Anoikis, a unique form of programmed cell death, is triggered when cells detach from adjacent cells or the extracellular matrix they typically adhere to. This mechanism is pivotal in the regulation of cellular survival, functioning by selectively eliminating cells that have abnormally detached from the adjacent structure^8^. Anoikis acts as a significant protective factor in both cancerous and non-cancerous diseases, particularly in inhibiting the dissemination of cancer cells that could lead to distant metastases^9^. Epithelial cells, which preserve normal tissue architecture through intercellular adhesion and engagement with the extracellular matrix, are particularly prone to anoikis^10^. Furthermore, epithelial cells constitute a critical component of tumor tissue. Tumor-Associated Epithelial Cells (TECs), in contrast to their normal counterparts, demonstrate resistance to anoikis, a critical precondition for tumor metastasis^11^. Anchorage-independent growth and the Epithelial-Mesenchymal Transition (EMT) are two pivotal hallmarks of anoikis resistance, significantly impacting tumor progression and the metastatic potential of cancer cells^12^. the activation of various signaling pathways plays a crucial role in imparting anoikis resistance to cancer cells, thereby enabling distant metastasis^13^. Extensive research has indicated that Anoikis-Related Genes (ARGs) are intricately linked with tumor progression and metastasis. The interaction between TIMP1 and CD63 activates the PI3K-AKT pathway, inducing resistance to anoikis in melanoma^14^, while CPT1A mediates fatty acid oxidation, promoting resistance to anoikis and inducing metastasis in colorectal cancer cells^15^.

The objective of this research is to investigate the regulatory impact of ARGs on ccRCC TECs at a single-cell level, and to establish an innovative risk prognostic model grounded on ARGs for ccRCC Anoikis-related epithelial subclusters (ARESs). Utilizing multiple datasets, the study validates the predictive efficacy of this model. Additionally, it develops a nomogram related to clinical features to augment its applicability in clinical settings. Additionally, this research examines the disparities in pathway and functional enrichment among patients from distinct risk groups, as well as the variance in immune cell infiltration within the TME grounded on gene signatures. The goal is to potentially pioneer novel approaches for diagnosing, prognostically evaluating, and tailoring treatments for ccRCC patients.

## Materials and methods

### Data collection and research design

Single-cell RNA sequencing (scRNA-seq) data were collected from tumor samples of seven patients with clear cell renal cell carcinoma (ccRCC) to explore the regulatory roles of ARGs in TECs. Additionally, spatial RNA sequencing (stRNA-seq) data from two ccRCC patients were downloaded to identify ARESs within the spatial context. We conducted thorough and extensive data mining to pinpoint robust DEGs between normal and tumor samples at the tissue level. The datasets included in this study adhered to the following criteria: each dataset was required to contain a minimum of 10 ccRCC tumor samples and corresponding adjacent normal samples. The entire scRNA-seq and stRNA-seq datasets, along with the microarray datasets, were obtained from the Gene Expression Omnibus (GEO) database, accessible at www.ncbi.nlm.nih.gov under the accession numbers GSE159115, GSE210041, GSE53757, GSE36895, GSE15641, GSE66272, GSE68417, and GSE40435. The bulk RNA sequencing data and clinical details for TCGA-KIRC were acquired from the TCGA database, accessible at https://cancergenome.nih.gov/. A supplementary validation dataset, E-MTAB-1980 was obtained from the ArrayExpress database, which can be accessed at https://www.ebi.ac.uk/arrayexpress/. All of the ARGs were retrieved from Gene Card database (https://www.genecards.org/), the protein coding genes and relevance score > 0.3 as filtering criteria. All data analyzed or generated in this study are freely accessible from prior publications or public databases.

### ccRCC scRNA-seq data processing

The 'Seurat' R package (version 4.4.0) was employed to preprocess the seven ccRCC scRNA-seq sample data. Seurat objects were generated for each sample, derived from their respective scRNA-seq gene expression matrix. Rigorous quality control was implemented, eliminating cells with gene expression counts below 200 or above 6000, raw counts under 1000, or mitochondrial gene expression surpassing 20%. Data normalization was subsequently carried out using the NormalizeData function, employing the LogNormalize method with its standard settings. The top 2000 variable genes were calculated using the FindVariableFeatures function. Employing these genes, the ScaleData and RunPCA functions were executed on the Seurat objects, extracting the top 30 principal components (PCs) for subsequent analysis. To remove batch effects across samples, the ‘Harmony’ R package was employed, followed by Uniform Manifold Approximation and Projection (UMAP) to visualize distinct cell clusters in each scRNA-seq dataset. Ultimately, major cell types within the ccRCC TME were annotated and visualized, leveraging ccRCC cell annotation data derived from previous studies^16^.

### Anoikis score and identify anoikis related TECs subcluster

The expression score for ARGs was calculated using normalized data from each cell cluster, employing the 'Ucell' R package. To investigate the regulatory impact of ARGs on TECs, the non-negative matrix factorization (NMF) algorithm was utilized, specifically version 0.26 of the NMF R package. This approach entailed conducting dimensionality reduction analysis on ARGs' expression in TECs, thereby categorizing distinct cell subtypes through the scRNA expression matrix. This analysis conformed to the methodologies outlined in previous relevant studies, guaranteeing consistency and comparability^17,18^. DEGs within each NMF subcluster were calculated via the FindAllMarkers function, which was employed with its standard parameters. Subclusters exhibiting an average log2 fold change (log2FC) in ARGs exceeding 1, and manifested expression in over 70% of cells, were classified as ARESs.

### Pseudotime analysis, cell communication, functional enrichment analysis and metabolic analysis of ARESs

The Monocle R package (version 2.30.0) was employed to analyze scRNA-seq data of ARESs, with the goal of elucidating the relationship between ARGs and cell pseudotime trajectories^19^. Dimensionality reduction via the DDRTree method enabled the visualization of ARGs' dynamic expression changes in ARECs' pseudotime trajectories in ccRCC, employing the plot_pseudotime_heatmap and plot_cell_trajectory functions for this purpose. Employing the Cellchat R package (version 2.1.0), tailored for scRNA-seq data analysis across various cell clusters, predictions of ARESs' cellular interactions were conducted using the CellChatDB.human database^20^. Subsequently, the circle plot was utilized to visualize the intensity and extent of intercellular communication networks among various ARESs. The 'netAnalysis_signalingRole_heatmap' function depicted the signaling pathway and their respective signaling input–output patterns. The scMetabolism function was applied to estimate cellular metabolism analysis, grounded on the related metabolic pathway enrichment results of ARESs’ DEGs. Finally, KEGG pathway enrichment analysis was performed on the DEGs between ARESs, employing the ClusterProfiler R package (version 4.10.0). Gene sets with an adjusted *p*-value less than 0.05 were categorized as having significant enrichment, and the top three pathways with the highest enrichment were showcased.

### Identifying ARESs in the spatial context of ccRCC patients

To identify within the spatial context and address the challenge of inter-patient heterogeneity, we utilized stRNA-seq data from two patients with ccRCC. The cell types identified at the single-cell level in our study served as references for spatial context. The Cell2location package, which employs a deep learning model to decode gene expression profiles from both scRNA-seq and stRNA-seq data to predict cell location and abundance^21^, was applied to deconvolute major cell types and ARESs in the stRNA-seq data. The pipeline and deconvolution process followed the original study and official documentation, with parameters set to default. Subsequently, we utilized the NMF algorithm to explore the colocalization relationships at the spatial level.

### Robust DEGs identification

The 'limma' R package was utilized to calculate DEGs between tumor tissue and adjacent normal tissue in six ccRCC GEO microarray datasets. Significantly upregulated and downregulated genes met these thresholds: adjusted *p*-value < 0.05 and |log2FC|> 1. A volcano plot was employed to illustrate the differential expression of these genes between normal and tumor groups. Subsequently, A systematical analysis was performed on DEGs extracted from a series of GEO ccRCC datasets, employing the robust rank aggregation (RRA) approach via the 'RobustRankAggreg' R package's aggregateRanks function. Genes that demonstrated a *p*-value lower than 0.05 were identified as robust DEGs specific to ccRCC.

### Development of a novel ARESs-related risk prognostic model

An intersection analysis was conducted among ARESs-related DEGs, ARGs, and robust DEGs to identify common genes. The identified common genes were considered as ARESs-related anoikis DEGs, which are implicated in the development and metastasis of ccRCC TECs. The TCGA-KIRC cohort underwent random partitioning into a training cohort and an internal validation cohort, adhering to a 7:3 distribution. Within the training set, all genes of interest were analyzed using survival-associated Lasso regression to optimize prognostic gene selection. Subsequently, the most pivotal genes underwent multivariate Cox regression analysis. For constructing the risk prognostic model, a threshold of a *p*-value under 0.05 was determined. A prognostic model for risk was constructed using gene expression levels and corresponding coefficients. The risk score was calculated in the following equation:

$$Risk score={\sum }_{i=0}^{n}coef \left({gene}_{i}\right)*Exp\left({gene}_{i}\right)$$

Using the median risk score as a criterion, samples from both the training and internal validation cohorts were categorized into ${risk}_{high}$ and ${risk}_{low}$ groups. Kaplan–Meier survival analysis was conducted to assess the correlation between overall survival duration and risk score. For assessing the accuracy of the prognostic model, the 'survivalROC' R package was employed to generate receiver operating characteristic (ROC) curve plots, focusing on the area under the curve (AUC). Finally, the E-MTAB-1980 dataset functioned as an external validation cohort, with the prognostic model's effectiveness evaluated using identical methodologies as previously described. To further estimate the prognostic efficiency of our risk model, we applied progression-free interval (PFI), disease-specific survival (DSS), and disease-free interval (DFI) analyses within the TCGA-KIRC cohort. Additionally, ccRCC patients were categorized into different subgroups based on various clinical characteristics (such as gender, age, TNM category, cancer stage, and grade) to assess prognostic potency at different levels of confounding factors.

### Development of prognostic nomogram

Both univariable and multivariable Cox regression analyses were applied to all clinical variables, aiming to pinpoint independent factors for prognosis, aiding in the development of a more effective nomogram. Employing the 'rms' R package, a comprehensive nomogram was developed, integrating the risk score with key clinical prognostic indicators. ccRCC patients in the TCGA-KIRC cohort were divided into ${nomo}_{high}$ and ${nomo}_{low}$ groups grounded on their cumulative nomogram scores. The predictive accuracy of the nomogram was evaluated using the ROC. Additionally, calibration curves for 1-, 3-, and 5-year predictions were generated to demonstrate the nomogram's prognostic capabilities. Ultimately, Decision Curve Analysis (DCA) for a 5-year period was utilized to elucidate the impact of each prognostic factor. Finally, the nomogram was validated in the E-MTAB-1980 cohort.

### Pathway and functional enrichment analysis

The 'GSVA' R package was used to calculate enrichment scores for hallmark gene pathways. Pathway activity disparities between the two risk groups were analyzed using the 'limma' R package. A significant difference was indicated by a |t-value|> 2 and a *p*-value < 0.05. The most recent hallmark gene set, h.all.v2023.2.Hs.symbols.gmt, was sourced from the Molecular Signatures Database (MSigDB). To identify DEGs between the risk groups using raw count data, the 'DESeq2' R package was deployed. Subsequently, all of the results were arranged in order of decreasing logFC and subjected to gene set enrichment analysis (GSEA) based on Gene Ontology (GO). Additionally, Spearman correlation analysis was conducted to identify genes associated with three key genes in the TCGA-KIRC cohort independently. All related genes were sorted in descending order of correlation coefficients and subjected to Gene Set Enrichment Analysis (GSEA) based on the 186 KEGG pathways^22–24^.

### Tumor microenvironment analyze based on risk prognostic model signatures

Risk prognostic model signatures were utilized to examine variations in the (Tumor Microenvironment) TME of ccRCC. The 'ESTIMATE' algorithm was utilized to determine the immune, stromal, and estimate scores, which serve as indicators of the level of immune and stromal cell infiltration, deduced from the gene expression profiles related to TME cells within the two risk groups. To obtain deeper insights, the 'CIBERSORT' and 'MCPcounter' R packages were applied, enabling a more detailed assessment of specific immune cell infiltration levels in each ccRCC patient. Employing Spearman correlation analysis, the interconnections among risk genes, the risk score, and various immune cells within the TME were evaluated. This integrated approach provided a detailed insight into the interaction between components of the TME and prognostic categories among patients.

### Exploring drug sensitivity in different risk group of ccRCC

The evaluation of drug efficacy, particularly through the half-maximal inhibitory concentration (IC50), is essential for determining drug sensitivity. Utilizing the 'oncoPredict' R package, the sensitivity of 198 drugs across two distinct risk groups was assessed. This analysis, focused on uncovering individualized treatment approaches, leveraged data from the Genomics of Drug Sensitivity in Cancer (GDSC) database. The box plot was employed to visually demonstrate the variances in drug sensitivity between these groups.

### Validation of prognostic genes in signature

To validate the gene expression and survival prognosis of the prognostic gene signature, we utilized the BEST (Biomarker Exploration of Solid Tumors) website^25^, which aggregates data from various public databases. The RNA expression of these three pivotal genes was assessed in different clinical groups. Subsequently, all genes underwent univariate Cox regression analysis across various ccRCC datasets to confirm their prognostic impact. Furthermore, for the assessment of gene expression at the protein level, the UALCAN website (https://ualcan.path.uab.edu/analysis-prot.html) was employed. This platform offers protein expression analysis utilizing data from both the Clinical Proteomic Tumor Analysis Consortium (CPTAC) and the International Cancer Proteogenome Consortium (ICPC) databases.

### Cell culture

Human renal cell lines HEK 293 and the ccRCC cell lines HRC-A498 and OS-RC-2, were purchase from the American Type Culture Collection (ATCC). The three cell lines underwent cultivation in Dulbecco's Modified Eagle Medium (DMEM, HyClone, GE Healthcare Life Sciences, USA), that containing 1% penicillin–streptomycin solution and 10% fetal bovine serum (FBS, HyClone, GE Healthcare Life Sciences, USA) at 37 °C in a humidified incubator with 5% CO2.

### RT‒PCR

For the isolation of total RNA, the MolPure® TRIeasy™ Plus Total RNA Kit (CatNo: 12309ES96) was utilized. Subsequent reverse transcription was performed with Hifair Biotechnology's BeyoRT II M-MLV transcriptase. Real-time PCR employed SYBR Green (CatNo: SY1020, Solarbio) and 2Taq PCR MasterMix (SR1110 Solarbio), conducted on an Exicycler 96 thermocycler (Solarbio). The analysis of the data was executed using the 2-∆∆Ct method, with glyceraldehyde-3-phosphate dehydrogenase (GAPDH) serving as an internal standard to normalize the expression levels of the target gene. The primer sequences were presented in Table S1.

### Statistical analysis

Data processing and statistical analyses in this study were conducted with R software (version 4.3.0). DEGs within each ARES were calculated using the Wilcoxon test. The survival disparities across different risk groups were assessed through Log-rank tests and Kaplan–Meier survival curves. To pinpoint critical prognostic genes and clinical variables, both lasso and multivariate Cox regression analyses were employed. Correlation coefficients were determined utilizing the Spearman method. A *p*-value less than 0.05 in a two-side test was deemed indicative of statistical significance in this research.

## Results

### Landscape of ccRCC cell clusters and anoikis status

**Figure 1:**
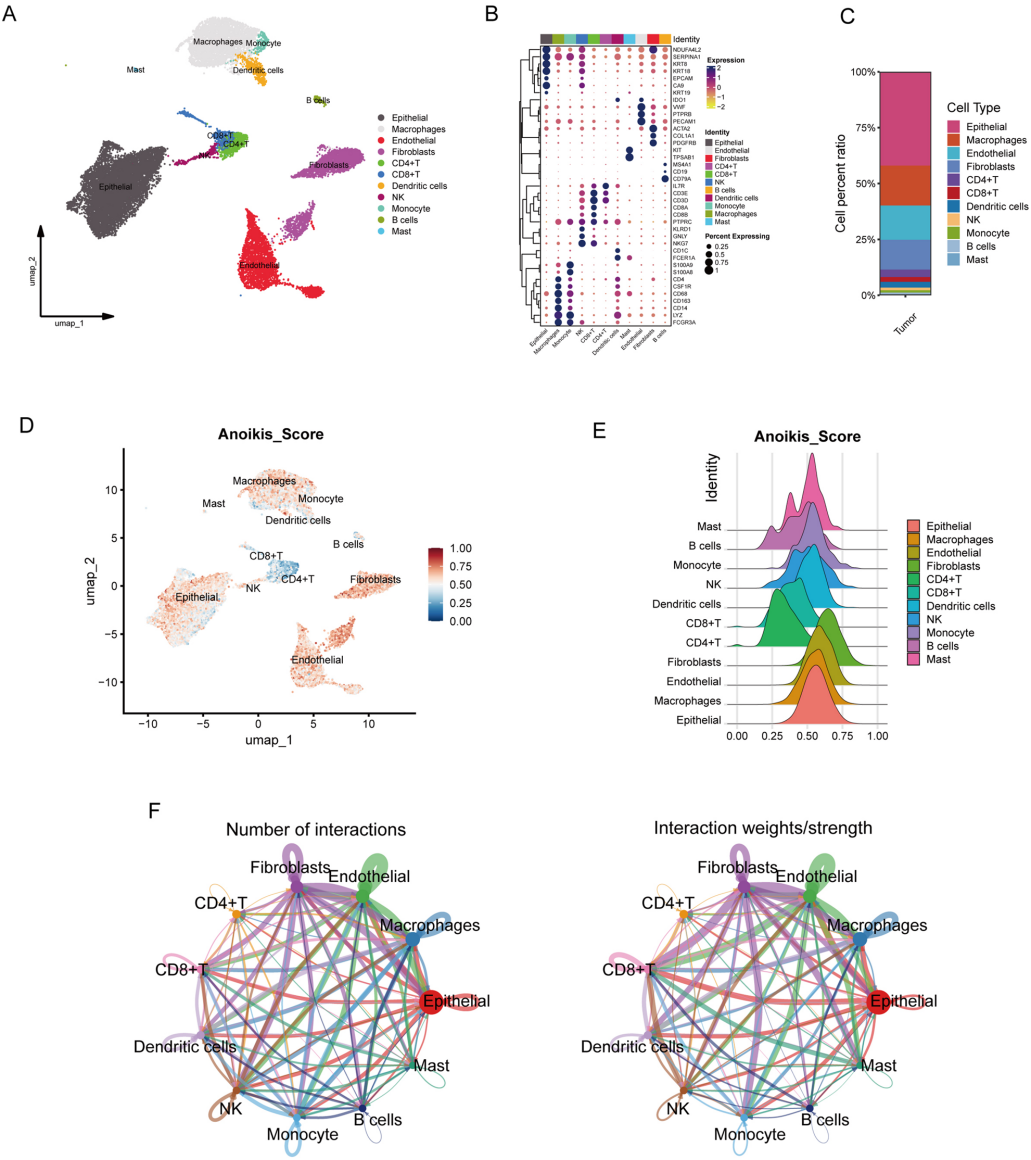
Cellular Landscape and Anoikis Status in ccRCC. (**A**) UMAP visualization representing 11 distinct cell types in ccRCC. (**B**) Distribution and gene expression profiles of marker genes across the identified cell types. (**C**) Proportion of the 11 major cell types within the ccRCC TME. (**D**) Anoikis scores for each cell type, calculated using ARGs via the UCell package. (**E**) Ridge plot depicting anoikis scores across the 11 major cell types. (**F**) Quantification and interaction strength among the 11 major cell types.

The workflow is presented in Fig. S1A. After strict cell quality control and the removal of unknown cells, a total of 22,978 single-cell data from ccRCC were retained. Subsequently, 11 major cell types within the TME of ccRCC were identified, based on previous studies in this field (Fig. 1A). The specific markers for these cell types are also presented in Fig. 1B. Significantly, disparities were observed in the proportions of these cell types, with epithelial cells constituting the largest proportion (Fig. 1C). Anoikis status in various cell types was evaluated using the AddModuleScore_UCell function, which calculated scores for eleven cell clusters based on a gene set of 541 ARGs (Table S2). Evidently, Epithelial cells, Macrophages, Endothelial cells, and Fibroblasts exhibited higher anoikis scores compared to other cell clusters within the ccRCC TME (Fig. 1D,E). To further explore cellular interactions, cell communication analysis was employed. This analysis revealed a diverse and intricate network of interactions among the cell clusters in the ccRCC TME (Fig. 1F). Particularly, TECs emerged as a pivotal component of the ccRCC cellular landscape and were thus selected for more detailed subsequent research.

### Comprehensive analysis of ARESs: classification, pseudotime, intercellular communication, pathway enrichment, metabolic activity and spatial location

**Figure 2:**
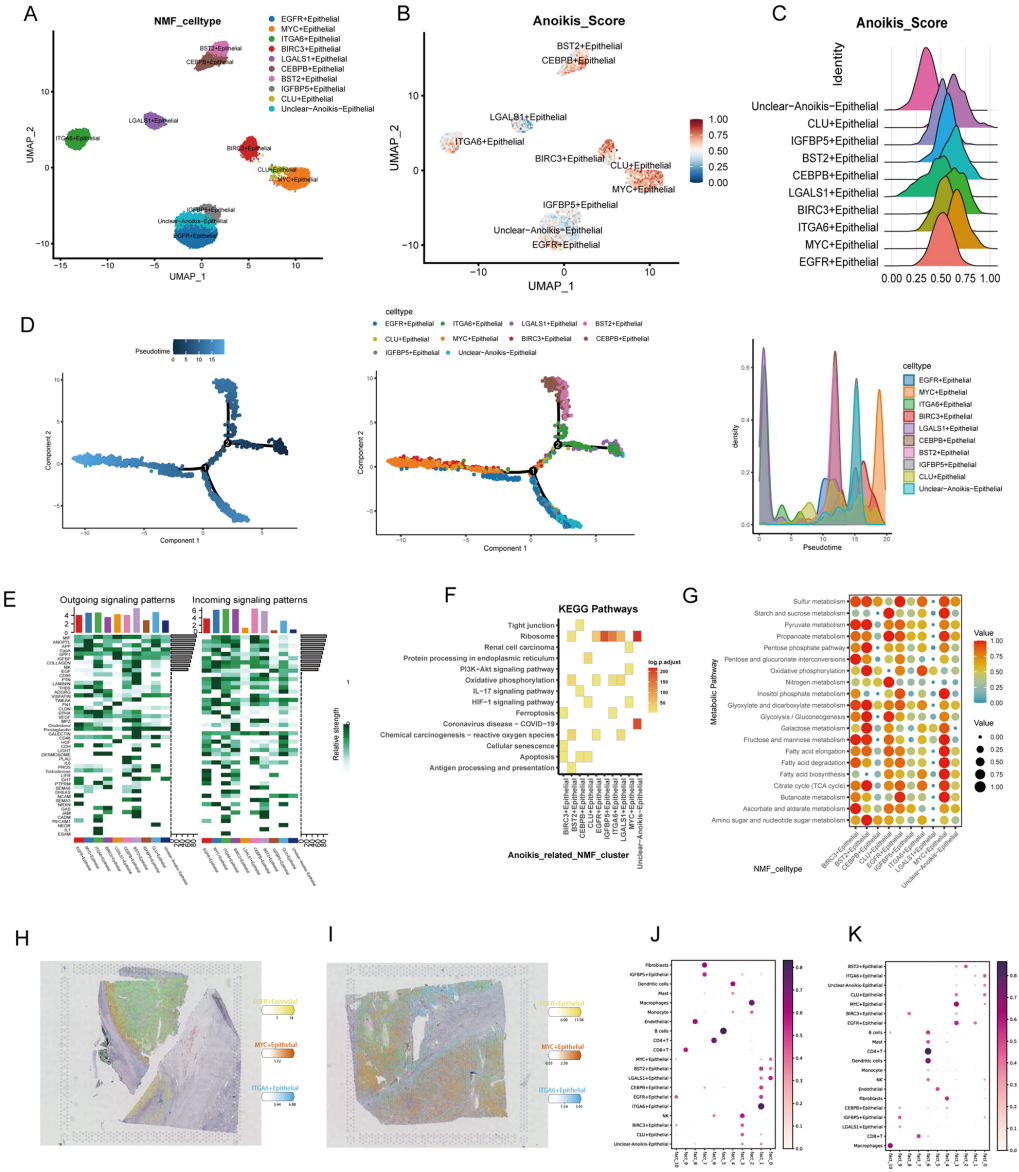
Comprehensive analysis of ARESs. (**A**) NMF analysis of TECs categorizes nine ARESs and identifies an unclear subcluster. (**B**) Comparison of anoikis status across different NMF subclusters. (**C**) Ridge plot illustrating anoikis scores across NMF subclusters. (**D**) Trajectory analysis revealing distinct differentiation patterns in the NMF subclusters of epithelial cells. Pseudotime analysis calculated using Monocle (left). Differentiation trajectory of NMF epithelial subclusters (middle). Cell density along the pseudotime (right). (**E**) Cell communication analysis illustrates diverse patterns of incoming and outgoing signaling. (**F**) KEGG pathway enrichment analysis highlighting significant pathway activation differences among epithelial subclusters. (**G**) Activity profiles of each subcluster in the top 20 metabolic pathways. (**H**-**I**) Spatial distribution of the top 3 abundant ARESs in HE sections from 2 ccRCC patients. (**J**-**K**) Dot plot illustrating the relationship of cell colocation based on the NMF algorithm.

Based on ARGs expression levels, TECs were categorized into 9 ARESs and an Unclear-Anoikis-Epithelial subcluster, as determined by the NMF algorithm (Fig. 2A). Employing the aforementioned method, the anoikis scores for all TEC NMF subclusters were calculated. The Unclear-Anoikis-Epithelial subcluster was observed to exhibit the lowest anoikis score among the subclusters (Fig. 2B,C). Pseudotime analysis revealed a dynamic temporal pattern in the expression of ARGs, significantly impacting the developmental trajectory of ARESs. Ultimately, the ARESs differentiated into MYC + epithelial subclusters, which exhibited the highest cell abundance at the end of pseudotime (Fig. 2D). Additionally, cell communication analysis revealed distinct receptor-ligand interactions among ARESs. Notably, the Unclear − Anoikis − Epithelial subcluster demonstrated weaker signaling activities compared to most of ARESs (Fig. 2E). KEGG enrichment analysis of DEGs within each ARES highlighted significant pathways including PI3K-Akt signaling pathway, Tight junction, Ribosome, Renal cell carcinoma, HIF-1 signaling pathway, Apoptosis, Ferroptosis among others (Fig. 2F). In terms of cellular metabolism, ARESs exhibited significant activity differences, with the Unclear-Anoikis-Epithelial subcluster showing relatively weaker metabolic activity (Fig. 2G).

### ARESs distribution at the spatial level

Our deconvolution analysis reveals that ARESs are identified within the spatial context of HE tissues sections of ccRCC. Figure 2H,I illustrates the top three cell abundances of ARESs in two patients, showing a similar colocalization pattern. Additionally, the localization results of each subcluster indicate the spatial specificity of ARESs (Fig. S2A-B). The NMF algorithm further demonstrated the colocalization relationships between ARESs and other major cell types in ccRCC. We observed that ARESs exhibit distinct colocalization states at the spatial level in different ccRCC patients, and that immune cells and stromal cells interact variously with ARESs (Fig. 2J,K).

### Differential expression of epithelial ARGs identified

**Figure 3:**
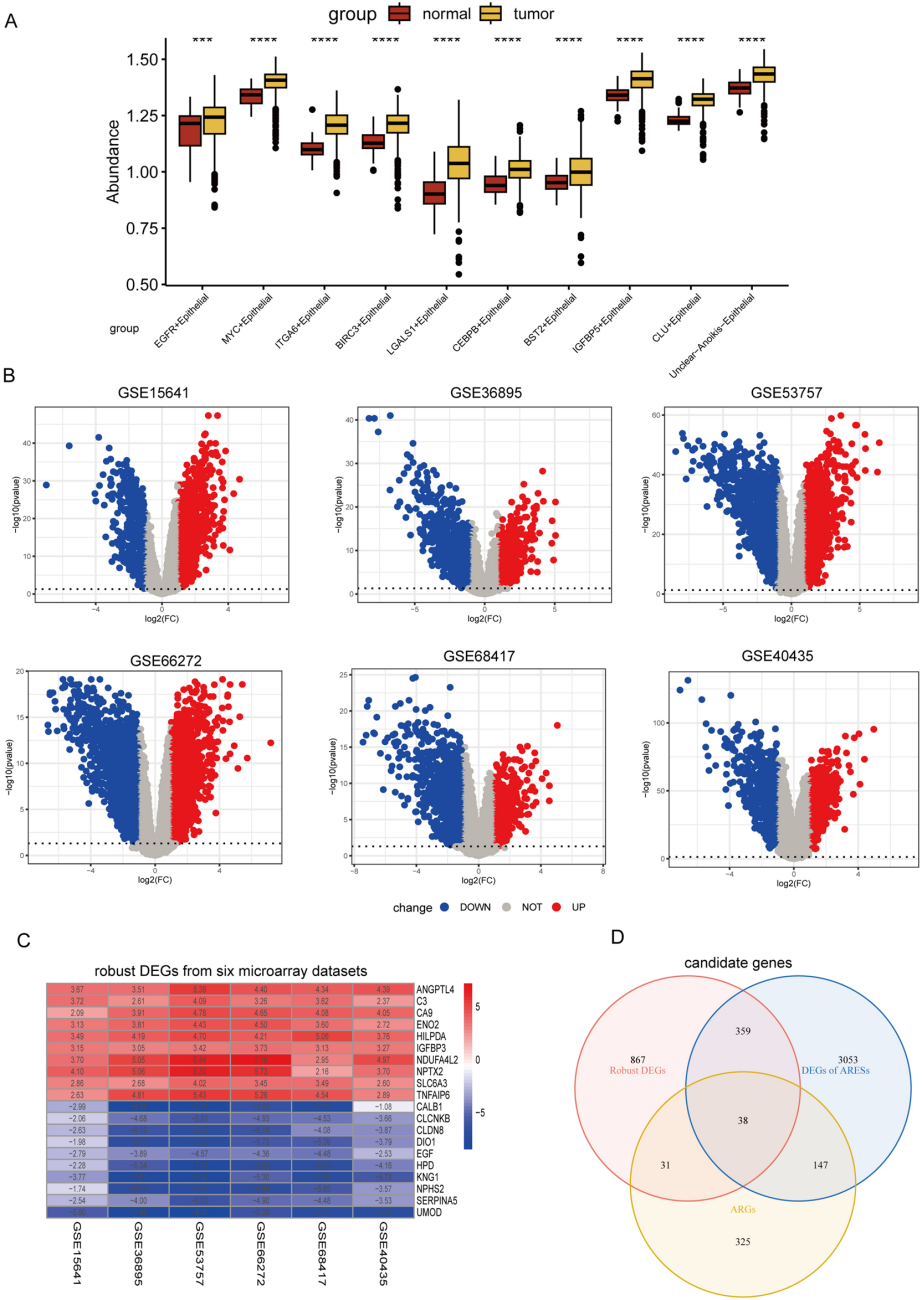
Differential gene expression and candidate gene identification in ccRCC. (**A**) DEGs of each ARES in tumor versus normal samples of the TCGA-KIRC cohort. (**B**) Volcano plot showing DEGs calculated by limma R package from GSE15641, GSE36895, GSE53757, GSE66272, GSE68417, and GSE40435 datasets. (**C**) The top 10 upregulated and downregulated robust DEGs calculated by RRA using DEGs from six GEO datasets. (**D**) Venn diagram showing the intersection of ARES DEGs, robust DEGs, and ARGs, yielding 38 candidate genes.

The FindAllMarkers function was employed to identify DEGs among various ARESs, yielding a total of 3597 DEGs (Table S3). Subsequently, these DEGs were analyzed for expression levels in the TCGA-KIRC cohort using bulk RNA data. The DEGs from each subcluster exhibited elevated expression in tumor tissues (Fig. 3A). Furthermore, employing six ccRCC microarray datasets (GSE53757, GSE36895, GSE15641, GSE66272, GSE68417, GSE40435), DEGs were calculated for each dataset, and RRA was utilized to identify a total of 1,295 robust DEGs (Fig. 3B and Table S4). The heatmap showed the top 10 upregulated and downregulated robust DEGs (Fig. 3C). The intersection of ARESs DEGs, robust DEGs from the six GEO cohorts, and ARGs yielded 38 candidate genes for prognostic analysis (Fig. 3D).

### Development and assessment of a novel ARESs-based risk prognosis model in ccRCC

**Figure 4:**
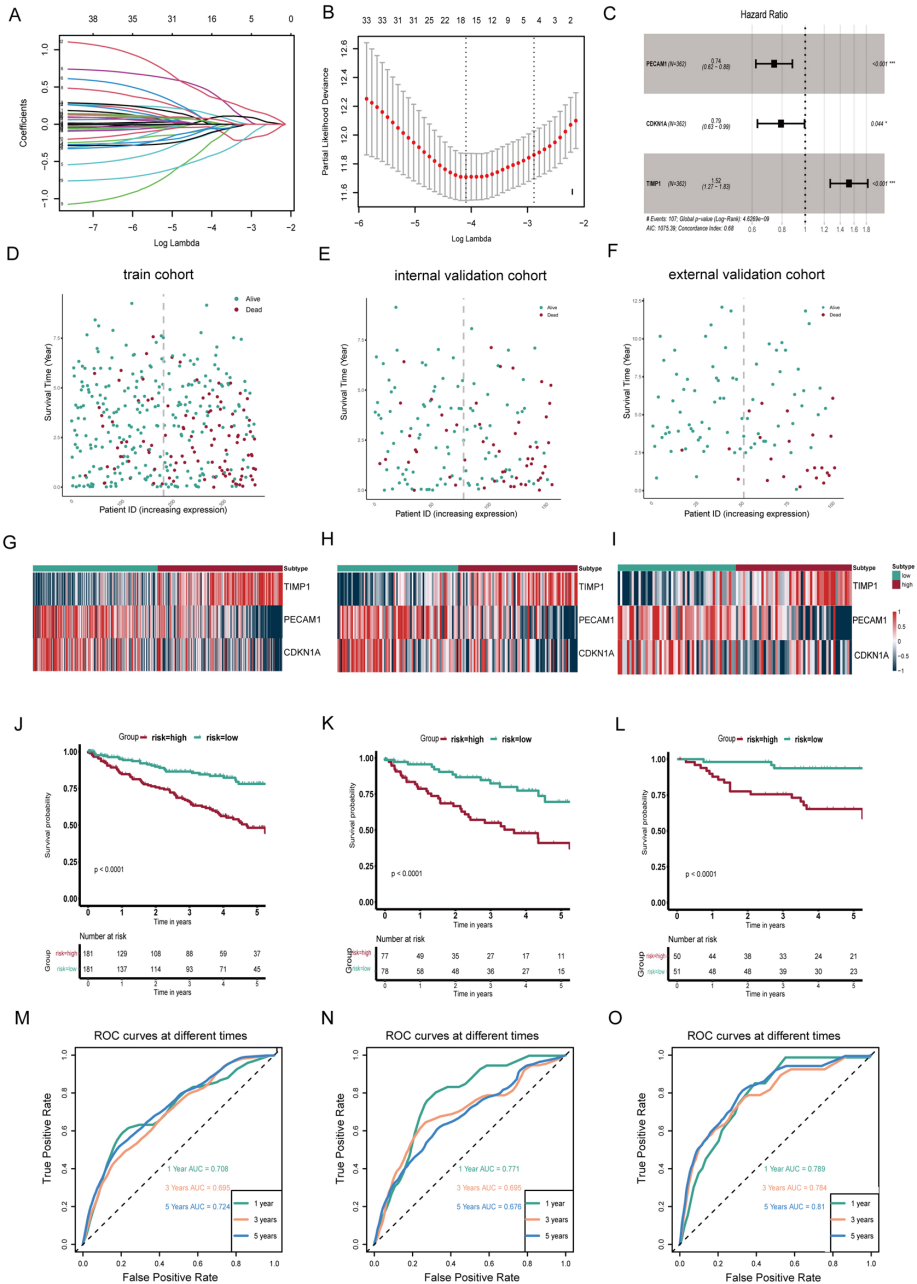
Construction and validation of an innovative ARESs-based risk prognosis model. (**A**-**B**) LASSO regression analysis applied to optimize prognostic genes in the TCGA training cohort. (**C**) Multifactorial Cox analysis of pivotal gene. (**D**-**F**) Distribution of patient status with increasing risk score in the training, internal validation, and external validation cohorts. (**G**-**I**) key genes expression in the two risk groups. (**J**-**L**) Kaplan–Meier survival curves for two risk groups (**M**–**O**) AUC of the prediction of 1, 3, and 5-year survival rates in the three cohort.

In the TCGA-KIRC cohort analysis involving 517 ccRCC patients, a random allocation into training (362 samples) and test (155 samples) cohorts was executed, adhering to a 7:3 ratio. LASSO regression, applied to the training cohort, highlighted four significant prognostic genes (CEBPB, PECAM1, CDKN1A, TIMP1) from 38 potential candidates (Fig. 4A,B). These genes then underwent multivariable Cox regression to establish their coefficients. Notably, TIMP1, PEACM1, and CDKN1A, each with a *p*-value below 0.05, were instrumental in developing the risk prognostic model. The risk score formulation was as follows:

$$\begin{aligned} {\text{Risk}}\;{\text{score}} = & \left( {0.{42114}*{\text{expression}}\;{\text{of}}\;{\text{TIMP1}}} \right) \\ & + \left( { - 0.{29888}*{\text{expression}}\;{\text{of}}\;{\text{PECAM1}}} \right) + \left( { - 0.{23282}*{\text{expression of CDKN1A}}} \right). \\ \end{aligned}$$

**Figure 5:**
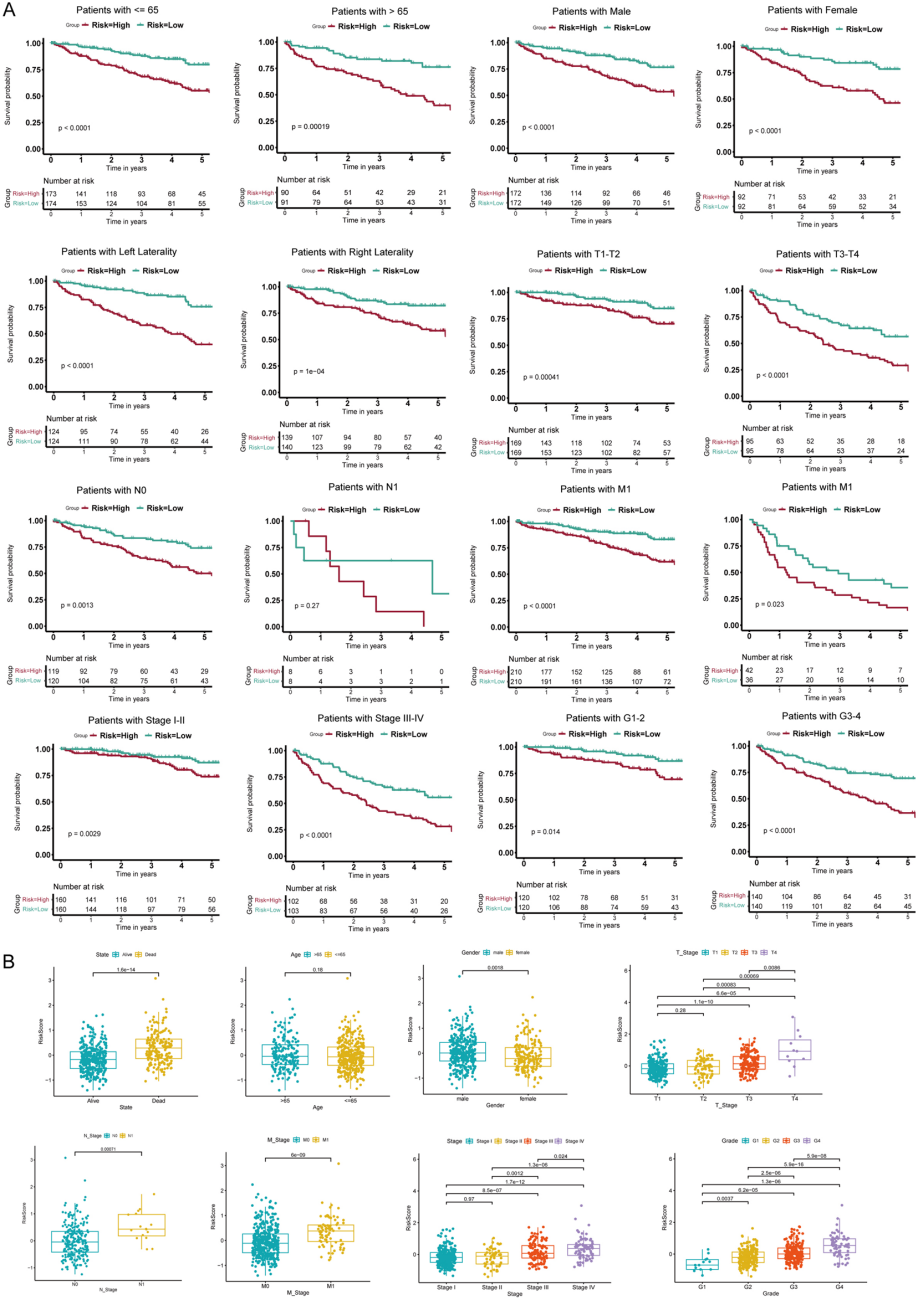
Relationship between risk score and clinical characteristics in ccRCC. (**A**) Kaplan–Meier survival curves for patients stratified by different clinical features, including age, gender, laterality, TNM category, cancer stage, and grade. (**B**) Box plots illustrating the distribution of risk scores across various clinical subgroups.

These genes, predominantly upregulated in tumor samples, played distinct roles: TIMP1 as an oncogene (HR > 1) and PECAM1 and CDKN1A as tumor suppressors (HR < 1), as depicted in Fig. 4C. The division of the training cohort into high-risk and low-risk groups was based on the median risk score. An increase in risk scores correlated with a higher mortality rate in the high-risk group (Fig. 4D–F). Notably, TIMP1 was highly expressed in the high-risk group, in contrast to PECAM1 and CDKN1A, which were more expressed in the low-risk group (Fig. 4G–I). Survival analysis revealed a less favorable prognosis for the high-risk group (Fig. 4J–L). The AUC values, indicating predictive accuracy for 1-, 3-, and 5-year survival rates, were 0.708, 0.695, and 0.724, respectively (Fig. 4M). Independent internal and external validations, performed in the test cohort and the E-MTAB-1980 dataset, yielded similar results (Fig. 4N,O). Additionally, all ccRCC patients in the TCGA-KIRC cohort were stratified into various subgroups based on clinical characteristics, including age, gender, laterality, TNM category, and stage. Survival analysis results consistently demonstrated the effectiveness of this risk model, confirming that it is not biased by potential confounding factors (Fig. 5A). Furthermore, risk scores were calculated for different subgroups. Box plots revealed that ccRCC patients who were deceased, male, had larger tumor masses, lymphatic metastasis, distant metastasis, advanced stage, and high grade, exhibited higher risk scores (Fig. 5B). These findings underscore that the risk score is significantly associated with tumor progression and metastasis. Ultimately, the risk prognostic model was further validated using the progression-free interval (PFI), disease-specific survival (DSS), and disease-free interval (DFI) in the TCGA-KIRC cohort (Fig. S3A-C). The findings demonstrate that this model exhibits superior prognostic efficacy across various prognostic indicators.

### Expression validation of prognostic genes in signature

**Figure 6:**
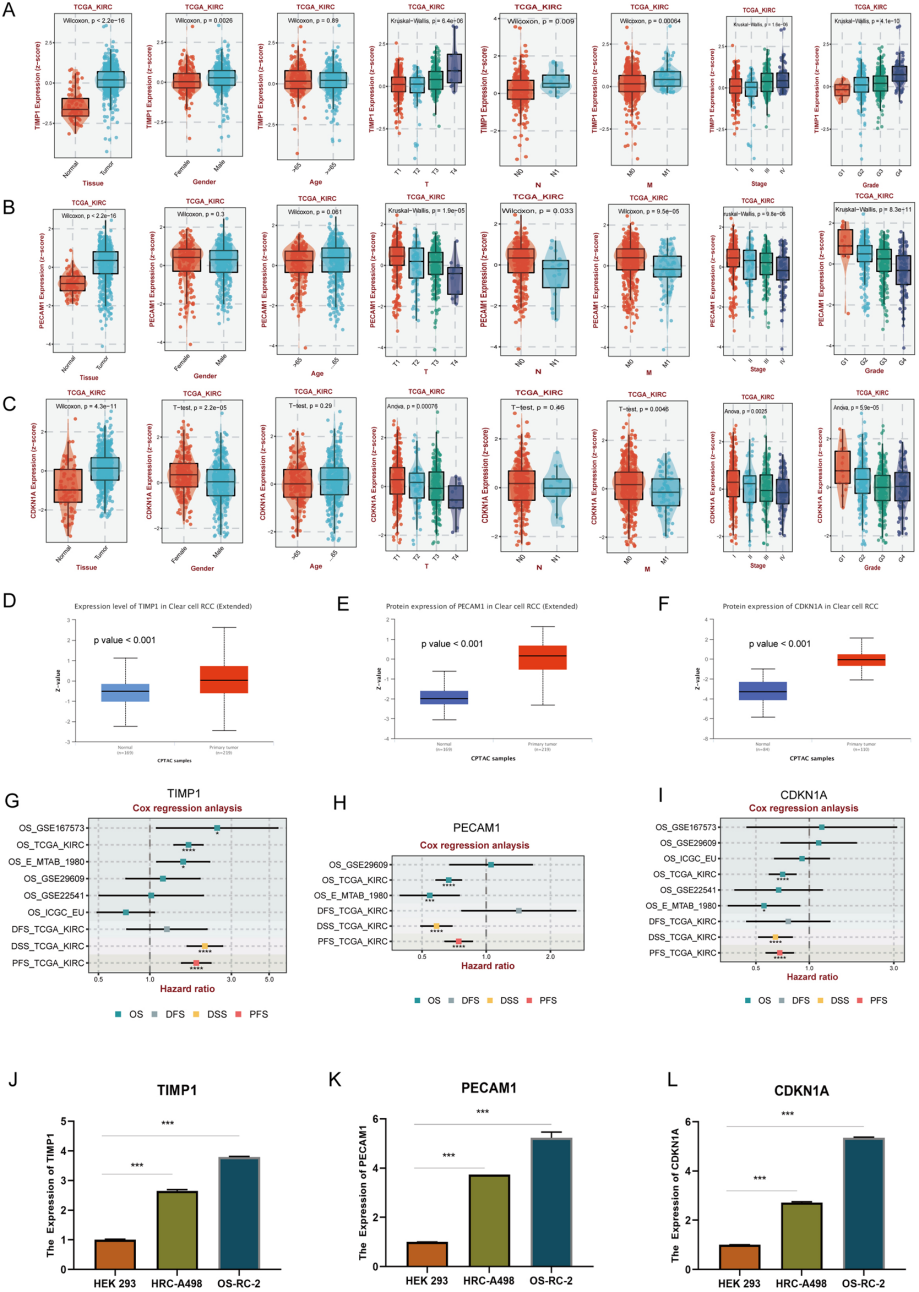
Validation of prognostic genes in signature. (**A**-**C**) mRNA expression levels of TIMP1, PECAM1, and CDKN1A validated using the BEST website across different clinical subgroups, including age, gender, laterality, TNM category, cancer stage, and grade. (**D**-**F**) Protein expression validation of TIMP1, PECAM1, and CDKN1A using the ULCAN website. (**G**-**I**) Univariate Cox regression analysis of TIMP1, PECAM1, and CDKN1A across various datasets. (**J**-**L**) Quantification of mRNA expression levels of TIMP1, PECAM1, and CDKN1A in HEK 293 and ccRCC cell lines (HRC-A498 and OS-RC-2) by RT-PCR. ***p < 0.001; **p < 0.01; *p < 0.05.

This study conducted a thorough validation of prognostic gene expression signatures at both mRNA and protein levels. Utilizing the BEST website, the RNA expression of three key genes was analyzed across various clinical groups. TIMP1 was found to be upregulated in tumor samples, and this elevated expression was associated with higher TNM categories, advanced cancer stages, and grades (Fig. 6A). Conversely, PECAM1 and CDKN1A were upregulated in tumor tissues but downregulated in groups with higher TNM categories, advanced cancer stages, and grades (Fig. 6B,C). Significantly, protein expression analysis via the UALCAN website, referencing CPTAC and ICPC databases, showed elevated protein levels of these genes in tumor tissues versus normal tissues, underscoring their role in cancer development (Fig. 6D–F). Furthermore, univariate Cox regression analysis demonstrated that the hazard ratios of three key genes were consistent with the coefficients in the risk model across various datasets (Fig. 6G–I). RT-PCR validation experiments confirmed a substantial upregulation of TIMP1, PECAM1, and CDKN1A mRNA in ccRCC tumor cell lines (HRC-A498, OS-RC-2) relative to a normal renal cell line (HEK 293), as illustrated in Fig. 6J–L.

### Development of prognostic nomogram

**Figure 7:**
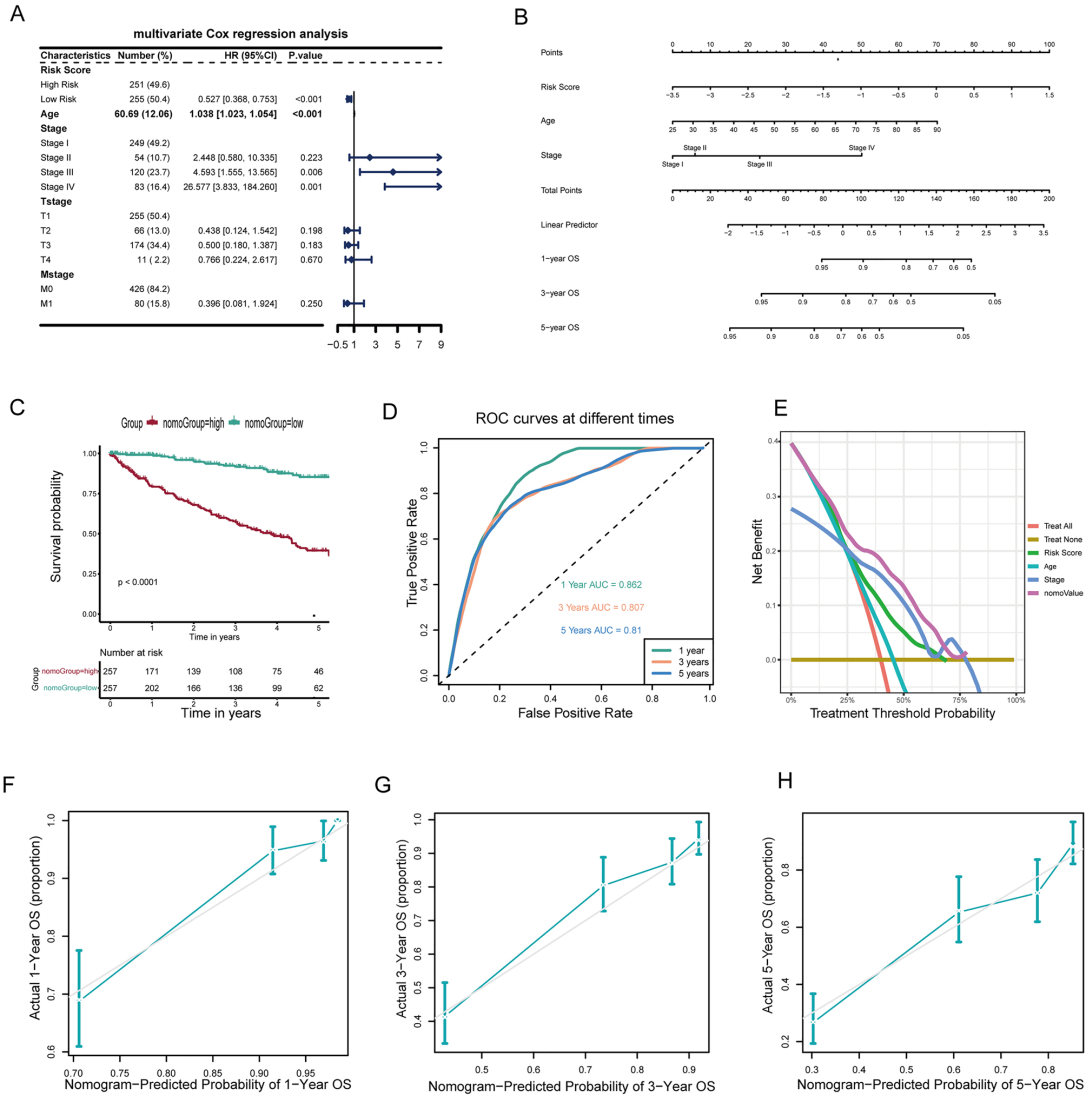
Development of prognostic nomogram. (**A**) Multifactorial Cox analysis of risk score and clinical features. (**B**) Combining age, stage, and risk score, a nomogram was established to quantitatively predict overall survival in ccRCC patients. (**C**) Kaplan–Meier survival curves for two nomogram groups. (**D**) AUC for predicting 1, 3, and 5-year survival rates in the TCGA-KIRC cohort. (**E**) DCA for a 5-year period. (**F**–**H**) Calibration curves for 1, 3, and 5 years effectively showcased the robust predictive ability of this nomogram.

In the TCGA-KIRC cohort study, both univariable and multivariable Cox regression analyses were conducted on prognostic clinical features and risk scores to determine independent prognostic factors. These results are presented in a forest plot (Fig. 7A). Subsequently, a nomogram integrating the risk score with two clinical features (age and stage) was constructed to improve clinical applicability (Fig. 7B). Patients in the ${nomo}_{high}$ group exhibited a notably poorer survival prognosis compared to another group (Fig. 7C). Regarding 1-, 3-, and 5-year survival predictions within the TCGA-KIRC cohort, the AUC values were determined to be 0.862, 0.807, and 0.810 respectively (Fig. 7D). DCA of 5-year period highlighted the significant contribution of various variables to prognostic prediction, with the risk score emerging as a particularly crucial determinant (Fig. 7E). Additionally, the calibration curves for 1, 3, and 5 years effectively demonstrated the model's robust predictive ability (Fig. 7F–H). Subsequently, the efficiency of the nomogram was assessed using the E-MTAB-1980 cohort, which exhibited prominent predictive accuracy (Fig. S3D-E).

### Differential pathway and functional enrichment analysis

**Figure 8:**
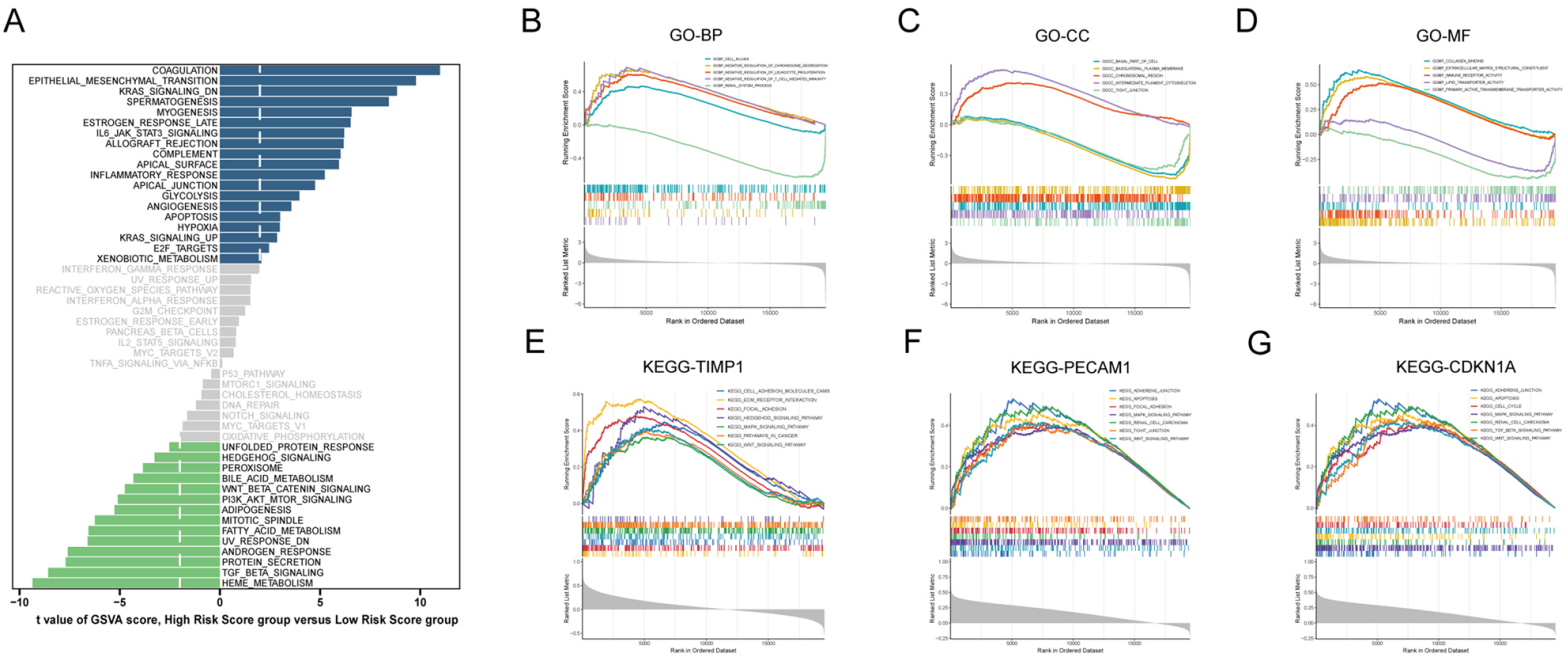
Differential pathway and functional enrichment analysis across distinct risk group. (**A**) Hallmark pathway enrichment results in the two risk groups using GSVA. (**B**-**D**) Gene Set Enrichment Analysis results for Cellular Component, Biological Process, and Molecular Function. (**E**–**G**) Correlated genes with TIMP1, PECAM1, and CDKN1A subjected to GSEA for KEGG pathways.

This study conducted a comprehensive pathway and functional enrichment analysis to distinguish between different risk groups. Employing the GSVA function revealed significant variations in the activation of hallmark signaling pathways between the two groups. Specifically, the ${risk}_{high}$ group demonstrated significant enrichment in pathways including Coagulation, Epithelial-Mesenchymal Transition (EMT), Inflammatory Response, Apoptosis, and Hypoxia. Conversely, the ${risk}_{low}$ group showed enhanced activation in PI3K AKT MTOR Signaling, TGF Beta Signaling and Wnt Beta Catenin Signaling pathways (Fig. 8A). Additionally, we performed Gene Ontology (GO) Enrichment Analysis using the GSEA function, utilizing DEGs between the two groups. This analysis underscored significant distinctions across Cellular Components (CC), Biological Processes (BP), and Molecular Functions (MF). Specifically, in BP, genes upregulated in the high-risk group were predominantly enriched in pathways such as negative regulation of chromosome segregation, leukocyte proliferation, and T-cell mediated immunity (Fig. 8B). Conversely, in CC, genes overexpressed in the low-risk group were primarily involved in tight junctions, the basolateral plasma membrane, and the basal part of the cell (Fig. 8C). Regarding MF, there was a notable correlation with collagen binding, structural constituents of the extracellular matrix, and immune receptor activity (Fig. 8D). Furthermore, we conducted Spearman correlation analysis to investigate gene functions, revealing that genes associated with three key markers showed enrichment in pathways critical for cell adhesion, the MAPK signaling pathway, renal cell carcinoma, and apoptosis, all significantly related to anoikis (Fig. 8E–G).

### Assessment of immune infiltration in the ccRCC TME

**Figure 9:**
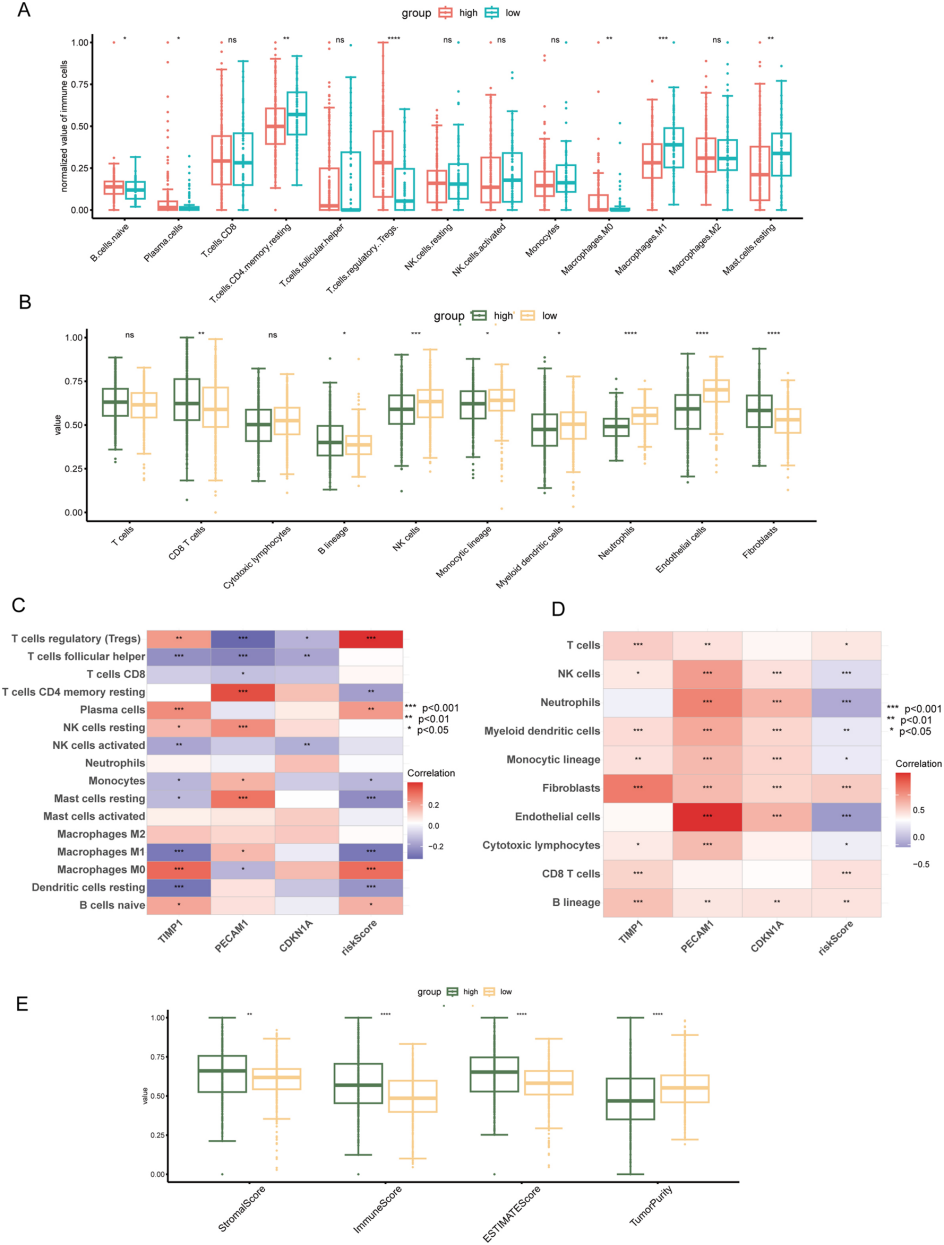
Assessment of immune infiltration in the ccRCC TME. (**A**) CIBERSORT analysis of immune cell infiltration in high-risk and low-risk groups. (**B**) MCPcounter analysis of major TME cell proportions in different groups. (**C**-**D**) Correlation analysis between the three pivotal genes, risk scores, and immune cells, calculated separately using CIBERSORT and MCPcounter algorithms. (**E**) Comparison of immune microenvironment scores between high-risk and low-risk groups.

To assess immune cell composition within the ccRCC TME, the 'CIBERSORT' and 'MCPcounter' algorithms were utilized. These analyses revealed distinct immune cell distributions: M1 macrophages, CD4 + T cells, NK cells, mast cells, neutrophils, and endothelial cells were more prevalent in the low-risk group, while the high-risk group was characterized by an increased presence of Tregs, B cells, CD8 + T cells and fibroblast (Fig. 9A,B). Further, Spearman correlation analysis explored the relationship between three prognostic genes, the risk score, and immune cell presence in the TME. The analysis revealed a positive association between TIMP1, risk score, and the prevalence of Tregs, plasma cells, and fibroblasts, while showing a negative correlation with M1 macrophages. Similarly, PECAM1 and CDKN1A exhibited correlations with endothelial cells, fibroblasts, NK cells, and B cells (Fig. 9C,D). Broadly, using the ESTIMATE algorithm, the ${risk}_{high}$ group was found to have elevated immune and stromal scores and a lower tumor purity compared to the counterpart, indicative of immunologically active tumors (Fig. 9E).

### Comparative analysis of drug sensitivity in risk subgroups

**Figure 10:**
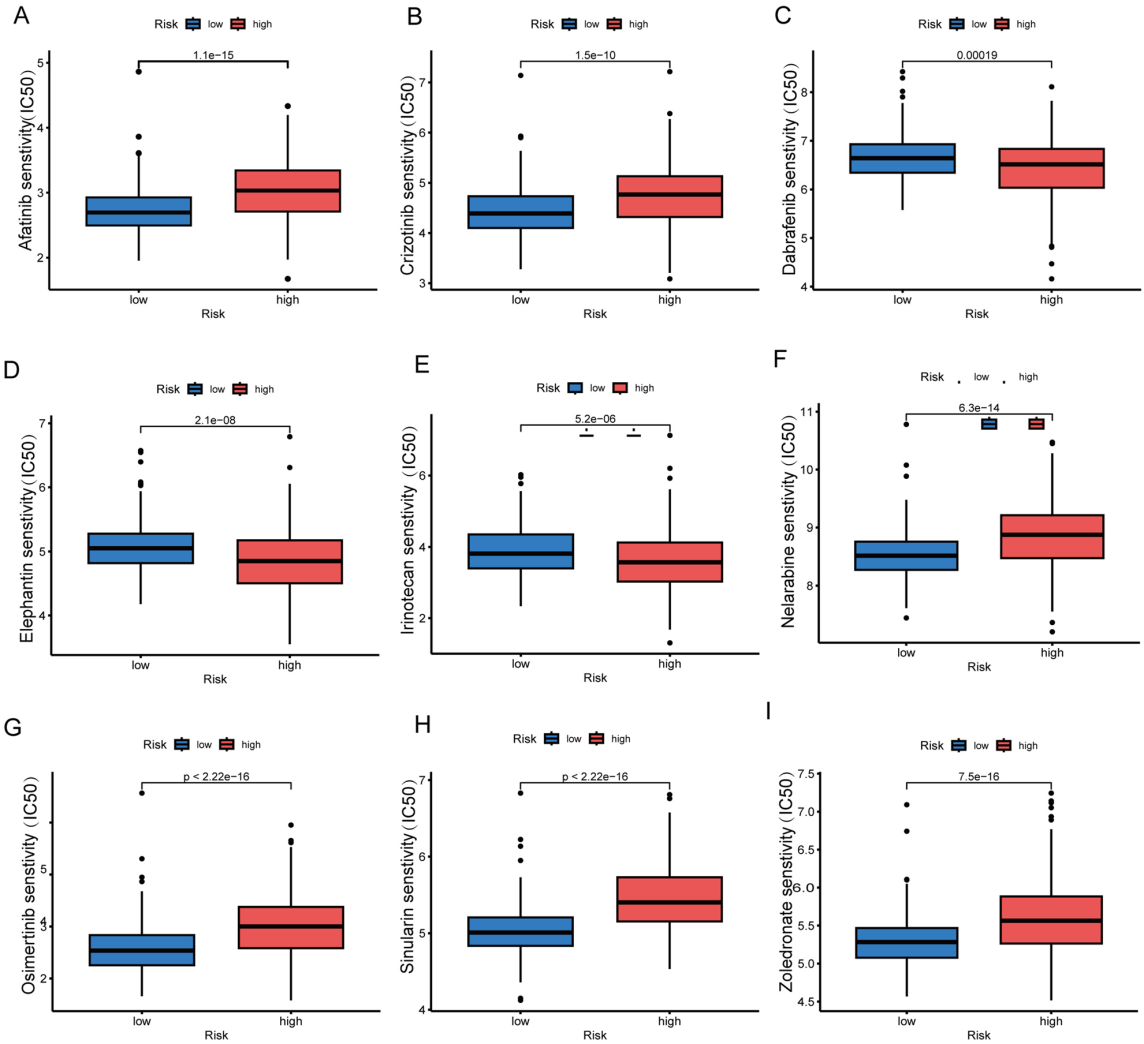
Comparative analysis of drug sensitivity in risk subgroups. (**A**-**I**) Box plots illustrating variations in drug sensitivity for nine drugs between two groups.

Drug sensitivity analysis revealed varying antineoplastic drug responses between the two risk groups. A box plot illustrates the variations in drug sensitivity across nine drugs between these groups (Fig. 10A–I). Notably, patients within the ${risk}_{low}$ group showed reduced IC50 values, indicating greater sensitivity to Afatinib, Crizotinib, Nelarabine, Osimertinib, Sinularin, and Zoledronate. Conversely, the ${risk}_{high}$ group displayed increased sensitivity to Dabrafenib, Elephantin, and Irinotecan in comparison to another group. These observations highlight the importance of customizing antineoplastic treatments based on risk stratification in clinical practices.

## Discussion

Despite advancements in multimodal therapeutic approaches for ccRCC, including surgical resection, chemotherapy, immunomodulatory therapies, and molecularly targeted agents, clinical outcomes are often compromised by intrinsic tumor heterogeneity. This heterogeneity often results in unsatisfactory responses and induces patients to distant metastasis, significantly elevating mortality rates associated with ccRCC^26^. Urologists encounter considerable challenges in improving the prognosis of ccRCC patients, with post-surgical survival rates for individuals with lymph node metastases ranging between 20%-30%. Moreover, acquired resistance to targeted therapeutics often emerges within the first year of treatment, further reducing long-term survival prospects^27,28^. Current biomarkers for ccRCC, while numerous, show limitations in predictive accuracy, lack robust external validation, and have limited utility in guiding clinical decision-making for diagnostic, prognostic, and personalized therapeutic strategies^29–31^. Consequently, there is a pressing requirement to devise and validate novel biomarkers and predictive models that can enhance the precision of prognostic evaluations and inform targeted treatment modalities in ccRCC management.

Anoikis, due to its unique mechanism, plays a pivotal role in tumor development and the spread of distant metastasis. Its initiation mainly involves the interaction between intrinsic pathways (disruption of mitochondrial functions) and extrinsic pathways (stimulation of cell surface death receptors). To resist anoikis and achieve metastatic dissemination, cancer cells adopt diverse strategies including selectively switching integrin profiles, undergoing EMT, continuously activating survival signaling pathways, and modifying energetic metabolism^11,32^. Furthermore, cancer cells can promote anoikis resistance through various stimulation such as hypoxic conditions, stromal cell influence, oncogenic activation, reactive oxygen species, and kinase activation, thereby enhancing their invasive and metastatic potential. Within the context of anoikis resistance, the PI3K/Akt signaling axis is identified as a principal pathway, with PI3K/Akt, MEK, and ERK serving as key regulators of this resistance mechanism^12,32^. Studies indicate that quinazoline-based drugs specifically target survival signals at RCC focal adhesions, inducing anoikis for therapeutic purposes^33^. However, the regulatory mechanisms exerted by ARGs in ccRCC tumor epithelial cells remain unclear. This study conducts a comprehensive single-cell RNA analysis of ARGs in ccRCC tumor epithelial cells, aiming to uncover key molecular insights. And further identify the distribution of ARESs based on the stRNA-seq analysis. By integrating these analyses with bulk RNA transcriptomics, the aim is to construct an innovative and effective risk prognosis model based on DEGs associated with ARESs, thereby elucidating the prognostic landscape of ARGs in ccRCC.

In this study, relevant ARESs were identified by analyzing ARGs expression across various TECs NMF subclusters. Furthermore, Pseudotime analysis revealed significant dynamic changes in ARGs expression within ccRCC ARESs, substantially influencing the developmental trajectory of these subclusters. In the end of differentiated trajectory, MYC + Epithelial subcluster had the highest cell abundance. Cell communication analysis uncovered extensive and diverse intercellular interactions among ARESs, with ARGs exerting significant differential effects on the signaling inputs and outputs between subclusters. Notably, the Unclear-Anoikis-Epithelial subcluster displayed significantly fewer input and output signals compared to other ARESs. Regarding KEGG pathway enrichment analysis, distinct differences were observed among ARES clusters. DEGs within the MYC + Epithelial subcluster showed significant enrichment in pathways including Renal Cell Carcinoma, PI3K-Akt, and HIF-1 signaling. Extensive research has demonstrated the PI3K-Akt pathway's involvement in promoting anoikis resistance across various disease^34–36^. Additionally, the study conducted by Rohwer and colleagues demonstrated that HIF-1α contributes to anoikis resistance by reducing the expression of α5 integrin^37^. It is evident that TECs in this cluster, under hypoxic conditions, activate the HIF-1 and PI3K/AKT pathways, leading to anoikis resistance. Furthermore, several ARESs demonstrated significant enrichment in the Ribosome and Oxidative Phosphorylation pathways, indicating marked activation of cellular metabolic pathways. ccRCC is considered a metabolic malignancy that undergoes metabolic reprogramming to survive in hypoxic conditions and evade immunosurveillance. This includes alterations in aerobic glycolysis, tryptophan, glutamine, arginine, and fatty acid metabolism^38^. Transcriptomics and metabolomics have emerged as predominant methods for identifying genetic and metabolic characteristics at the molecular level, as well as potential therapeutic targets^39^.Consequently, a metabolic analysis of ARESs was conducted, revealing significant activation and pathway activity variations, particularly in the MYC + Epithelial subcluster. previous research has shown that cancer cells resist anoikis by modifying their metabolic processes. The Warburg effect, in particular, has been noted for aiding anoikis resistance and encouraging the spread of distant metastasis^40^. HIF-1 promotes Warburg metabolism by transcriptionally regulating glycolytic enzymes^11^. Notably, metabolic reprogramming contributes to anoikis resistance in ccRCC TECs. Our findings indicate that the MYC + epithelial subcluster shows greater anoikis resistance compared to other subclusters, and that anoikis resistance progressively increases across all subclusters as the tumor advances.

An innovative prognostic model specific to ccRCC and related to ARESs has been constructed, incorporating three key ARGs: TIMP1 (Tissue Inhibitor of Metalloproteinases 1), PECAM1 (Platelet and Endothelial Cell Adhesion Molecule 1), and CDKN1A (Cyclin Dependent Kinase Inhibitor 1A). Pathway analysis revealed that these genes are positively correlated with cell adhesion, focal adhesion, ECM-receptor interaction, and tight junctions. Notably, all of these pathways are associated with cell interaction and adhesion, which not only provide physical attachment but also activate downstream signaling pathways such as PI3K/AKT, MAPK, and ERK^9^. These signaling pathways significantly impact the processes of anoikis occurrence and resistance^41,42^. Additionally, the MAPK and WNT signaling pathways were significantly enriched. Various in vitro experiments have demonstrated that sustained p38 MAPK activation can induce anoikis in epithelial and cancer cell lines^43–45^. The activation of the WNT signaling pathway and EMT-related changes in cell adhesion are crucial factors in tumor progression and metastasis^46^. Furthermore, PECAM1 and CDKN1A are involved in apoptosis pathways. Since anoikis is a special form of apoptosis, this may explain their protective roles in our model. Extensive research has highlighted the critical role of these genes in tumor progression. TIMP1, through its interaction with beta-1 and CD63, activates the PI3K-AKT pathway, thereby mediating anoikis resistance in melanoma and facilitating tumor progression in ccRCC^14,47^. Additionally, Hou’s team had validated knock-downing TIMP1 can suppress ccRCC cell migration and metastasis^47^. PECAM1, upon phosphorylation during cellular aggregation, promotes proliferation by inducing anoikis resistance in tumor cells^48^. CDKN1A, as a primary effector of the tumor suppressor P53, significantly influences tumor drug resistance^49^. Inhibiting LSD1 can reducing demethylation of CDKN1A gene promoter to restrain ccRCC cell growth^50^. The model exhibited robust prognostic accuracy, validated both internally, externally and across different prognostic indictors. In an independent external validation cohort, the model identified a high-risk group with significantly higher mortality, characterized by elevated expression of the oncogene TIMP1 and reduced expression of tumor suppressors PECAM1 and CDKN1A, correlating with a shorter overall survival. The model achieved impressive AUC values of 78.9%, 78.4%, and 81% for 1, 3, and 5 years, respectively, in external validation cohort. The results were consistent across training, internal, and external validation sets. To improve clinical utility, a nomogram integrating the risk signature with clinically significant features was constructed. It exhibited outstanding predictive accuracy, with AUC values of 0.862, 0.807, and 0.81 for 1, 3, and 5-year forecasts, respectively. DCA clarified how the risk signature, coupled with clinical features, enhances the model's predictive capacity. This underscores the practical significance of the risk prognostic model in assessing the prognosis of patients with ccRCC.

In the TCGA-KIRC cohort, patients were classified into high and low-risk categories using the median risk score. Pathway analysis indicated marked enrichment of EMT, hypoxia, IL6 JAK STAT3 signaling, apical junction, and angiogenesis pathways in the high-risk group. Conversely, the low-risk group showed substantial enrichment in PI3K AKT MTOR signaling and TGF Beta signaling pathways. Previous studies have showed that TGF beta1 mediated the inhibition of JAK2/STAT3 and PI3K/AKT signaling pathways via SH2B3, reducing lung cancer anoikis resistance and suppressing cancer cell proliferation, migration, and EMT^51^. The characteristic of cancer cells, including metabolic reprogram and limitless replicative potency and sustained angiogenesis, that induce hypoxia microenvironment formation^52^. Hypoxia can mediate EMT of cancer cells to promote occurrence of anoikis resistance^11^. Above all, patients within the high-risk group exhibit enhanced anoikis resistance, significantly impacting survival outcomes when compared to the low-risk group.

RCC is notorious for its immunogenic nature, being one of the most immune-infiltrated tumors across various cancer types^53^. The features of the TME profoundly influence biological and pathological processes, thereby affecting the response to systemic therapy^53–55^. It often creating an immunosuppressive TME by promoting the proliferation of immunosuppressive cells like T regulatory cells and myeloid-derived suppressor cells, which consequently hampers immune functionality^56^. Addressing the challenge of eliciting effective immune responses in RCC patients for immunotherapy, thereby delaying tumor progression and improving survival outcomes, remains a critical question^57^. Advanced algorithms, MCPcounter and CIBERSORT, were employed to thoroughly analyze immune cell infiltration in the TME of two risk groups in ccRCC. CIBERSORT revealed a pronounced elevation in Tregs and M0 macrophages, along with a reduction in M1 macrophages, CD4 + T cells, and mast cells in the high-risk group's TME. Correspondingly, MCPcounter results indicated a higher presence of NK cells, myeloid cells, neutrophils, and endothelial cells, and a reduced presence of fibroblasts in the TME of the low-risk group. Notably, Increased infiltration of Treg cells in the TME is consistently associated to poorer survival outcomes in multiple cancer types. This correlation is largely due to the role of Tregs in inhibiting effective anti-tumor immune responses^58^. The reduced infiltration of M1 macrophages, which primarily exert anti-tumor immune responses, contributes to an immunosuppressive TME^59^. CD4 + T cells can target tumor cells directly through cytolytic mechanisms or indirectly by modulating the TME and mediating CD8 + cytotoxic T lymphocytes to kill tumor cells^60^. Recently, a pan-cancer atlas of neutrophils revealed 10 distinct cell states of neutrophils and identified a subset of antigen-presenting neutrophils that can enhance immunotherapy and fine-tune the TME^61^. Additionally, stromal cells are important element of TME. Cancer-associated fibroblasts (CAFs) are key stromal components that secrete growth factors, inflammatory ligands, and ECM proteins, promoting tumor proliferation, therapy resistance, and immune exclusion^62^. While traditionally viewed as tumor-promoting, some studies targeting the Hedgehog signaling pathway in CAFs suggest they may occasionally exhibit tumor-restraining functions under specific conditions^63^. When endothelial cells acquire resistance to anoikis, their stromal functions change, facilitating the distant metastasis of tumor cells^34^. Although stromal cells do not induce anoikis resistance in tumor cells, they indirectly promote tumor progression and metastasis. Correlation analysis revealed a substantial positive relationship between the risk score and Tregs and fibroblasts, while showing a significant negative association with M1 macrophages, CD4 + T cells, neutrophils, and endothelial cells. This finding underscores the gene signature's ability to effectively categorize patients into high- and low-risk groups, distinguishing the characteristics of their immune microenvironments and tumor-surrounding stromal components. Apparently, ARGs can modulate the composition and distribution of immune and stromal cells that impact the systemic therapy and prognosis of ccRCC patients. Understanding the modulation mechanisms of the TME can guide tumor treatment^64^. The immunosuppressive milieu observed in the high-risk group is associated with diminished survival prospects, implying that patients in this category might derive greater benefit from immunotherapeutic interventions. For example, TKIs can mediate VEGF to regulate Tregs infiltration levels^65^. Finally, drug sensitivity analysis was performed for different groups to develop effective treatment strategies, revealing that patients in various groups exhibit sensitivity differences to multiple drugs, including afatinib, crizotinib, and dabrafenib.

This study investigated the regulatory impact of ARGs within TECs of ccRCC. The development of an innovative prognostic risk prediction model, grounded in three pivotal ARGs related to ARESs, has not only efficiently forecasted patient’s overall survival but also between prognostic genes, risk scores, and the extent of immune cell infiltration level within the TME. Additionally, the model facilitates the prediction of drug response in different risk groups, providing essential insights for clinical treatment strategies. Despite the promising findings, this study has several limitations, including a limited sample size and the need for more extensive external validation. Additionally, further in vitro and in vivo functional experiments are required to confirm the roles of key genes in anoikis resistance and to develop targeted therapies for patients exhibiting high anoikis resistance. These shortcomings will be addressed in our future research.

## Conclusions

This study, with its unique single-cell and spatial-level perspective, comprehensively investigated the complex regulatory network of ARGs in ccRCC. At the bulk RNA level, the risk prognostic model and nomogram were constructed and validated, confirming their efficacy in prognosis assessment. This study not only enhances the understanding of the biological characteristics of ccRCC but also offers new strategies and tools for clinicians in managing, prognosticating, and developing personalized treatment plans for ccRCC patients.

### Supplementary Information

Supplementary Information 1.

Supplementary Figures.

Supplementary Table S1.

Supplementary Table S2.

Supplementary Table S3.

Supplementary Table S4.

## Data Availability

All datasets utilized and/or analyzed in this study are publicly accessible via open access repositories. The specific datasets can be found in the Gene Expression Omnibus (GEO, https://www.ncbi.nlm.nih.gov/geo/), with accession numbers GSE159115, GSE210041, GSE53757, GSE36895, GSE15641, GSE66272, GSE68417, and GSE40435. Additionally, data from The Cancer Genome Atlas (TCGA, https://cancergenome.nih.gov/) and ArrayExpress (https://www.ebi.ac.uk/arrayexpress/).
