# Supplementary material for: Deciphering anoikis resistance and identifying prognostic biomarkers in clear cell renal cell carcinoma epithelial cells
Source: Sci Rep. 2024 May 27;14:12044. doi: 10.1038/s41598-024-62978-0 (PMC11130322; doi:10.1038/s41598-024-62978-0)
Supplement: Supplementary file 1 — Supplementary Information 1. [file 41598_2024_62978_MOESM1_ESM.docx]

**Deciphering anoikis resistance and identifying prognostic biomarkers in clear cell renal cell carcinoma epithelial cells**

**Supplementary materials**

Supplementary Figure S1. Workflow of the Study. (A) Diagram illustrating the overall workflow and methodology used in this study.

Supplementary Figure S2. Deconvolution Analysis on the Spatial Context Based on scRNA-seq Data. (A-B) Distribution of 10 NMF epithelial subclusters on the HE section of ccRCC patient 1 and 2. Brighter spot colors indicate higher predicted probabilities of the corresponding cell types.

Supplementary Figure S3. Predictive efficiency validation of risk model and nomogram. (A-C) Validation of the risk model on different prognostic indicators, including Progression-Free Interval (PFI), Disease-Specific Survival (DSS), and Disease-Free Interval (DFI) in the TCGA-KIRC cohort. (D) Overall Survival Kaplan-Meier analysis of the nomogram in the E-MTAB-1980 cohort. (E) Receiver Operating Characteristic curve of the nomogram for OS in the E-MTAB-1980 cohort.

Supplementary Table S1. Sequences of Primers Used in RT-PCR. List of primer sequences utilized for real-time PCR in this study.

Supplementary Table S2. Anoikis-Related Genes. List of 541 anoikis-related genes analyzed in this study.

Supplementary Table S3. Differentially Expressed Genes (DEGs) Across 10 NMF Epithelial Subclusters. Comprehensive list of all DEGs identified across the 10 NMF epithelial subclusters.

Supplementary Table S4. Robust DEGs Calculated by Robust Rank Aggregation. List of robust DEGs identified using the RRA method based on six array RNA datasets, including GSE53757, GSE36895, GSE15641, GSE66272, GSE68417, and GSE40435.
