# Supplementary figures and images for: Deciphering anoikis resistance and identifying prognostic biomarkers in clear cell renal cell carcinoma epithelial cells
Source: Sci Rep. 2024 May 27;14:12044. doi: 10.1038/s41598-024-62978-0 (PMC11130322; doi:10.1038/s41598-024-62978-0)

Fig. S1

A

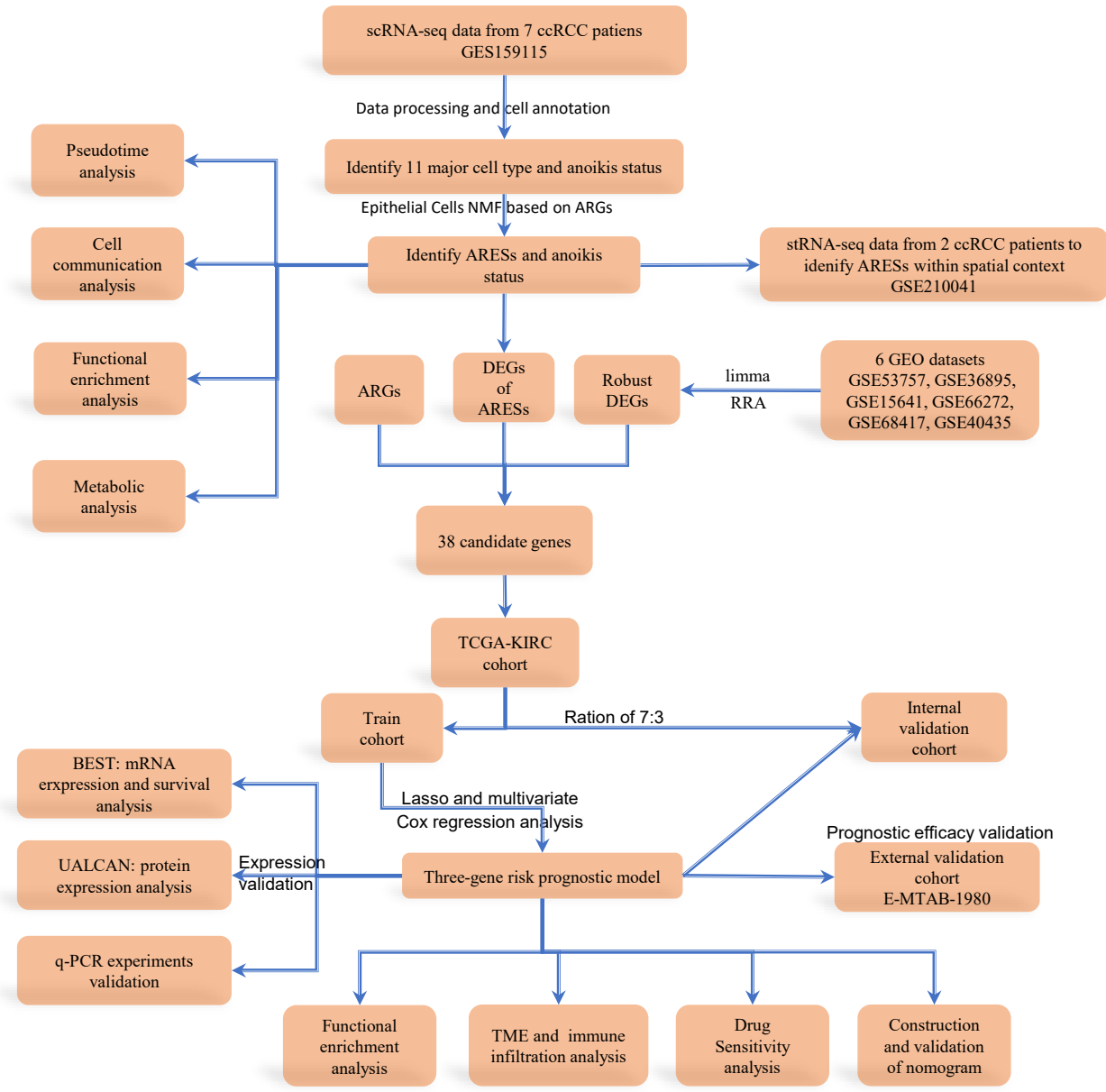

Fig. S2

A

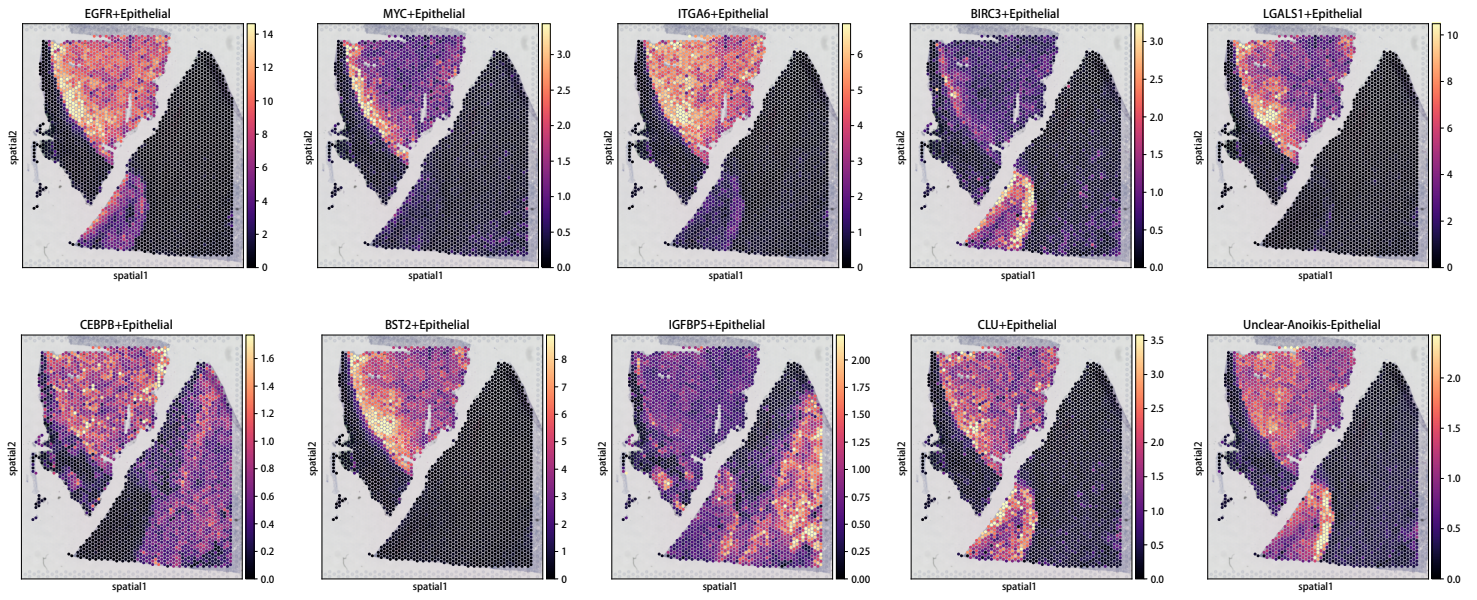

B

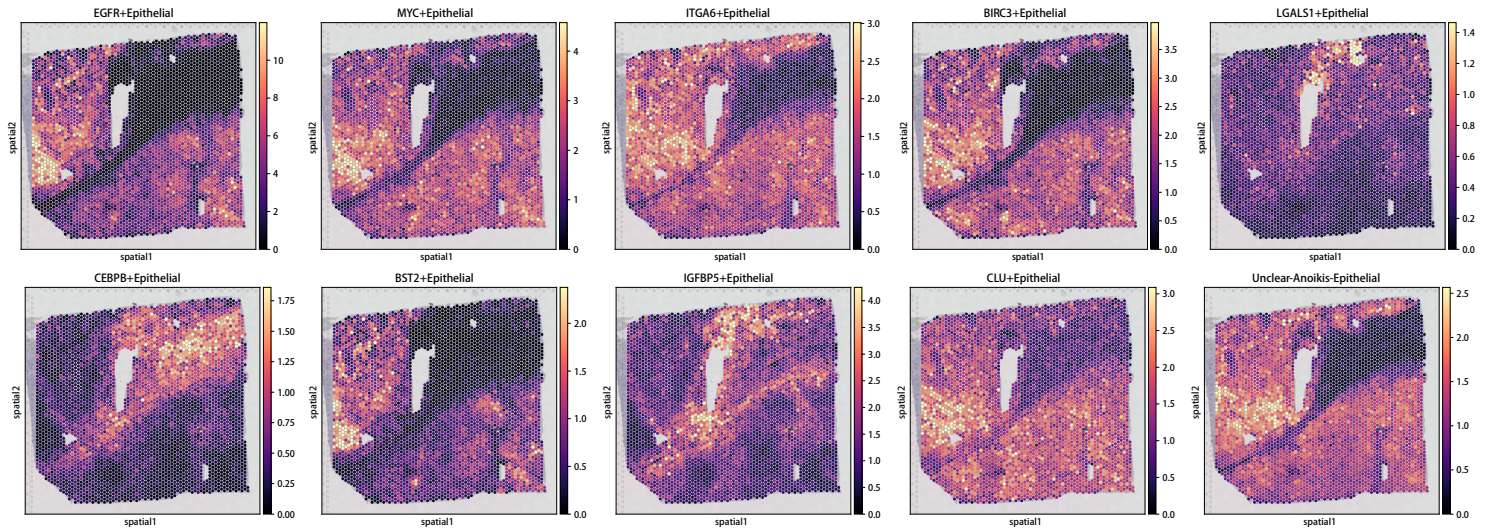

Fig. S3

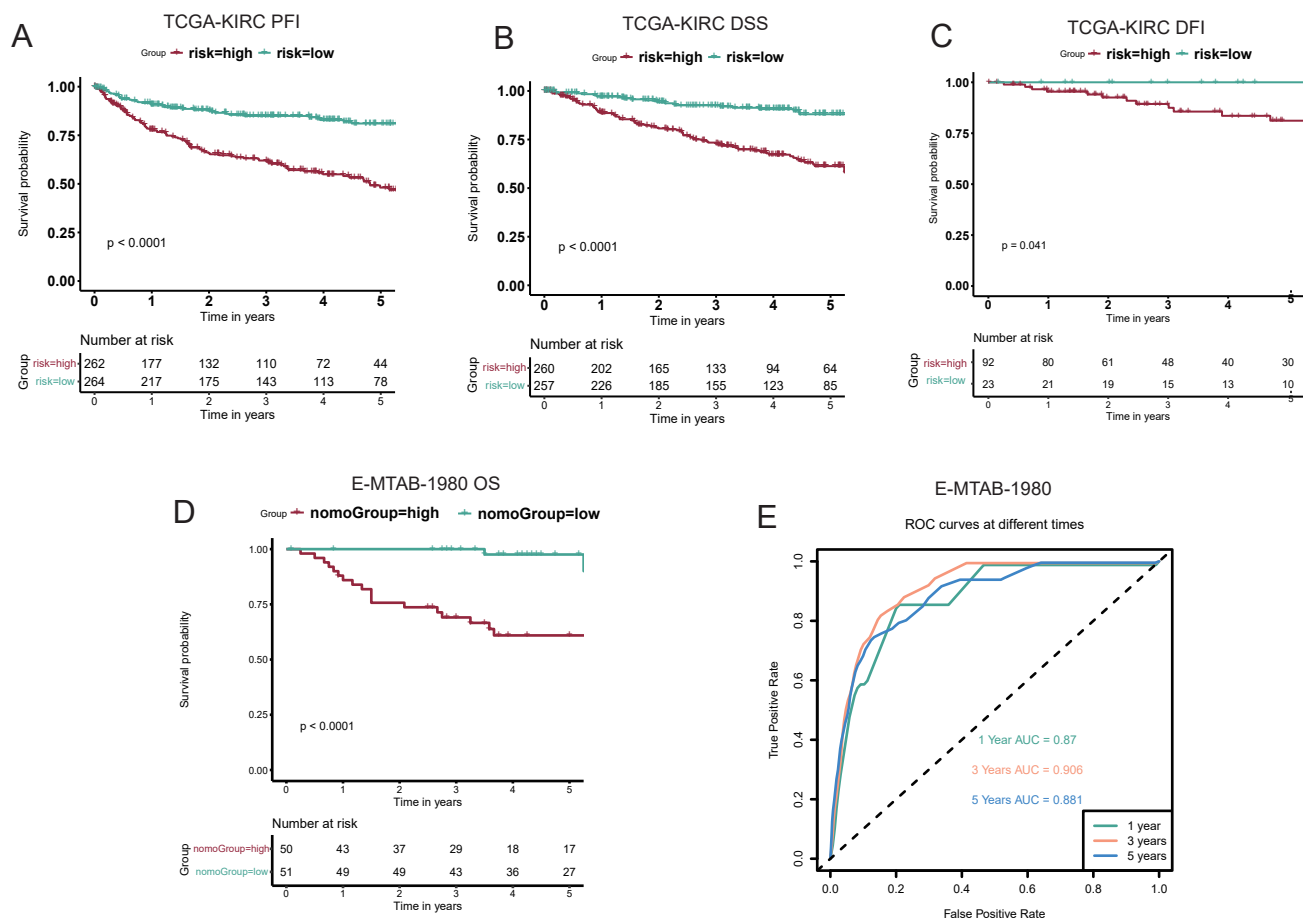

Supplement: Supplementary file 2 — Supplementary Figures. [file 41598_2024_62978_MOESM2_ESM.pdf]
